# Divergent Genomic Drivers in Benign-Appearing Lung Precursors and Their Synchronous Carcinomas

**DOI:** 10.3390/cancers18111691

**Published:** 2026-05-22

**Authors:** Jieun Lee, Yuchae Jung, Seung Yun Lee, Ye Won Song, Jongsun Jung, Chan Kwon Park, Young Jo Sa, Tae-Jung Kim

**Affiliations:** 1 Department of Hospital Pathology, Yeouido St. Mary’s Hospital, College of Medicine, The Catholic University of Korea, Seoul 07345, Republic of Korea; jieun.lee@catholic.ac.kr (J.L.); seungyun7680@gmail.com (S.Y.L.); 2 Department of Artificial Intelligence Convergence & Engineering, Open Cyber University of Korea, Seoul 02087, Republic of Korea; ycjung7@ocu.ac.kr; 3 College of Medicine, The Catholic University of Korea, 222 Banpo-daero, Seocho-gu, Seoul 06591, Republic of Korea; angelinaasdf@naver.com; 4 Syntekabio, Genome Data Integration Center, 519, Techno World 2, No. 187, Yuseong-gu, Daejeon 34025, Republic of Korea; jung@syntekabio.com; 5 Division of Pulmonology, Department of Internal Medicine, Yeouido St. Mary’s Hospital, College of Medicine, The Catholic University of Korea, 10, 63-ro, Yeongdeungpo-gu, Seoul 07345, Republic of Korea; ckpaul@catholic.ac.kr; 6 Department of Thoracic Surgery, Yeouido St. Mary’s Hospital, College of Medicine, The Catholic University of Korea, 10, 63-ro, Yeongdeungpo-gu, Seoul 07345, Republic of Korea; sagoon@catholic.ac.kr

**Keywords:** lung adenocarcinoma, squamous cell carcinoma, atypical adenomatous hyperplasia, squamous dysplasia, whole-exome sequencing, clonal evolution, EGFR, TP53, U2AF1, hallmarks of cancer

## Abstract

Background/Objectives: How the histologically benign tier of lung preinvasive lesions—atypical adenomatous hyperplasia (AAH) and squamous dysplasia (SD)—relates genomically to its paired carcinoma is unclear. To identify early versus late events, we compared synchronous preinvasive and invasive lesions from the same patient. Methods: Whole-exome sequencing was performed on 33 FFPE samples from 11 patients (7 AAH–lung adenocarcinoma [LUAD] and 4 SD–squamous cell carcinoma [SqCC] pairs, with paired normal lung). FFPE artefacts were mitigated by paired-normal subtraction, panel-of-normals filtering, and orthogonal caller cross-validation. Cancer-panel variants were classified as cancer-only, shared, or preinvasive-only. Results: Only ∼10% of cancer-panel variants were shared between paired lesions (∼50% carcinoma-only, ∼40% preinvasive-only), indicating that benign AAH/SD do not broadly mirror the paired carcinoma. Within this small shared fraction, the early-driver pattern diverged between tracks: AAH–LUAD pairs tended to share *EGFR* alterations, whereas the four SD–SqCC pairs featured *MET*-pathway alterations without any shared *EGFR* events. *TP53* and most other canonical drivers were carcinoma-confined (within-cohort contrast direction-consistent but non-significant); three patients lacked any canonical driver despite substantial mutational burden. Conclusions: In this pilot cohort, benign AAH and SD were genomically largely distinct from their paired carcinomas, sharing only a small set of key drivers whose identity diverged between glandular and squamous tracks. This suggests that benign-appearing AAH/SD differ from the more advanced AIS/MIA precursors not only histologically but also at the genomic level. These hypothesis-generating findings require confirmation in larger, multi-omic cohorts.

## Simple Summary

Lung cancer is the leading cause of cancer-related death worldwide, and many lung cancers begin as small, non-invasive growths called atypical adenomatous hyperplasia or squamous dysplasia. Why some of these early lesions develop into invasive cancer while others do not is poorly understood. In this study, we sequenced the protein-coding regions of the genome in 33 tissue samples from 11 patients, comparing each patient’s matched normal lung, early lesion, and invasive cancer. We identified which genetic changes are already present in the early lesion, which arise only in the invasive cancer, and which are shared. Mutations in the EGFR gene appeared early and were shared between lesions, whereas mutations in TP53, KRAS, and other cancer-driving genes typically appeared only in the invasive cancer. These findings help clarify the genetic steps that convert an early lung lesion into an invasive tumour and may inform future early detection strategies.

## 1. Introduction

Lung cancer remains the leading cause of cancer-related death worldwide, with non-small cell lung cancer (NSCLC) accounting for approximately 85% of cases and 5-year survival remaining below 25% largely because most cases are diagnosed at an advanced, locally invasive or metastatic stage [1,2,3]. The two major NSCLC histologies—lung adenocarcinoma (LUAD) and squamous cell carcinoma (SqCC)—differ markedly in their etiology, anatomic distribution, and molecular landscape: LUAD arises peripherally and is enriched for activating *EGFR* alterations in never-smokers, whereas SqCC arises centrally, is strongly tobacco-associated, and is dominated by complex copy-number alterations and near-universal *TP53* mutation rather than by single-gene oncogenic drivers [2,3,4]. Both lineages are thought to arise through histologically defined preinvasive stages, and it is important to emphasise that these stages are not a single category. Peripheral LUAD is thought to evolve from atypical adenomatous hyperplasia (AAH)—a small (≤5 mm) proliferation of mildly atypical pneumocytes that, like low-grade bronchial squamous dysplasia (SD) in the central airways, is classified as a histologically benign lesion regarded more as a probable forerunner of carcinoma than as a frank neoplasm [5,6,7]—and then progresses through adenocarcinoma in situ (AIS) and minimally invasive adenocarcinoma (MIA), which are categorised separately as in situ/minimally invasive carcinoma rather than as benign lesions [8]; the corresponding intermediate on the squamous track is carcinoma in situ (CIS) [6,7]. Despite their shared description as “preinvasive”, AAH/low-grade SD and AIS/MIA/CIS therefore occupy distinct classification tiers, and how closely the genomic content of the benign-appearing tier resembles that of the in situ/early-invasive tier and of frank carcinoma remains a key open question. Critically, longitudinal bronchoscopic studies have shown that most low-grade SD lesions regress or remain stable and that only a minority of AAH or SD lesions progress to invasive carcinoma [9,10], leaving a fundamental clinical problem unresolved: which histologically benign-appearing lesions are biologically committed to becoming cancer?

Beyond the cell-autonomous genomic events of the tumour cells themselves, the tumour microenvironment (TME)—comprising immune infiltrates, stromal fibroblasts, vasculature, and the surrounding airway epithelium—is now recognised as a decisive determinant of which preinvasive lesions progress and which regress. Premalignant AAH already shows Th1/Th2 imbalance and checkpoint upregulation [11], and a 280-gene airway field-of-injury transcriptomic signature can distinguish patients with versus without preinvasive lesions and even predict regression versus progression of bronchial dysplasia with high discriminative accuracy [6]. Together with evidence that immune-microenvironment remodelling, including HLA loss of heterozygosity, accompanies the preinvasive-to-invasive transition in LUAD [12], these observations argue that any molecular framework of early lung carcinogenesis must consider not only the tumour-cell genome but also the immune and stromal context in which it evolves.

Several large sequencing studies have begun to map the LUAD-side of this continuum. Multi-region whole-exome sequencing of 116 indeterminate pulmonary nodules [13] identified progressive variant accumulation and *EGFR*-mutant clonal sweeps; whole-exome and RNA sequencing of 98 AIS/MIA and 99 LUAD specimens [12] identified *TP53* mutation, arm-level copy-number alterations, and HLA loss of heterozygosity as the alterations most enriched in the invasive stage; and targeted sequencing of AAH/AIS/MIA samples reached concordant conclusions [11,14]. However, a critical gap remains: most of these studies profile preinvasive and invasive lesions either from different patients or as anatomically distinct nodules, and very few directly compare a preinvasive lesion to a synchronous invasive carcinoma arising from the same patient, on the same germline and shared environmental exposure. Moreover, the SqCC arm of the continuum—SD versus paired SqCC—has been even less systematically sequenced than the AAH–LUAD arm, leaving open whether the dominant early molecular events are shared between glandular and squamous pathways or fundamentally divergent.

The present study was designed to address this gap. By performing whole-exome sequencing on synchronous, microdissected preinvasive–invasive lesion pairs together with paired normal lung from the same patient, we directly compared the somatic mutational landscape of a benign-appearing precursor to that of its adjacent invasive carcinoma while controlling for inter-individual germline and exposure differences. The paired design enabled per-variant classification by variant allele frequency pattern into cancer-only, shared, and preinvasive-only events, providing a within-patient view of which alterations are early, persistent, or late. By analysing both AAH–LUAD and SD–SqCC pairs in parallel, we further asked whether the dominant early drivers are shared between the two histologic tracks or whether early lung neoplasia follows pathway-specific genomic trajectories that warrant separate biomarker frameworks.

## 2. Materials and Methods

### 2.1. Patients and Tissue Samples

Between 2010 and 2020, eleven patients who underwent lobectomy at Yeouido St. Mary’s Hospital (The Catholic University of Korea, Seoul, Republic of Korea) were enrolled in this study. All patients harboured a primary non-small cell lung carcinoma together with a histologically confirmed synchronous preinvasive lesion within the resected lobe. The cohort consisted of seven patients with lung adenocarcinoma accompanied by atypical adenomatous hyperplasia (AAH) and four patients with lung squamous cell carcinoma accompanied by bronchial squamous dysplasia (SD). Because AAH (≤5 mm) and SD are very small, morphologically subtle lesions that are frequently not apparent on gross inspection and often detected only as incidental microscopic findings, extensive histologic sampling of the resected lobectomy specimens—with careful mapping of the entire lobe—was required to reliably identify them adjacent to each invasive carcinoma, an approach previously applied to AAH characterization [11]. For each patient, three spatially distinct tissue regions were then sampled: histologically normal lung parenchyma (matched normal), the preinvasive lesion (AAH or SD), and the invasive carcinoma. All lesions were independently reviewed and confirmed by two board-certified pathologists according to the WHO classification of lung tumours [15]. A total of 33 samples (11 normal, 11 preinvasive, 11 carcinoma) were submitted for whole-exome sequencing.

### 2.2. Tissue Microdissection, DNA Extraction, and Quality Control

Formalin-fixed, paraffin-embedded (FFPE) tissue sections were serially cut and manually microdissected under a microscope using a water-moistened needle to enrich the target lesion area. Large carcinoma foci generally required only a few sections, whereas atypical adenomatous hyperplasia and squamous dysplasia often required more than 50 serial sections to obtain sufficient DNA for whole-exome sequencing, a strategy previously applied to similarly small preinvasive lesions [16,17]. Genomic DNA was extracted using the Covaris S220 focused-ultrasonicator (Covaris, Woburn, MA, USA) according to the manufacturer’s FFPE workflow [18]. DNA concentration and purity were assessed using a NanoDrop spectrophotometer (Thermo Fisher Scientific, Waltham, MA, USA) and the Qubit dsDNA HS Assay (Thermo Fisher Scientific). Whole-exome sequencing was performed only when at least 50 ng of genomic DNA was obtained, a threshold informed by prior work demonstrating reliable WES performance at low DNA input amounts [19].

### 2.3. Whole-Exome Library Preparation at the Broad Institute

Whole-exome libraries were constructed at the Broad Institute of MIT and Harvard (Cambridge, MA, USA) using a scalable, fully automated process for the generation of sequence-ready, hybrid-selection exome libraries, as previously described [4,20]. Briefly, ≥50 ng of genomic DNA from each microdissected FFPE sample was sheared to a target fragment size of ∼150–200 bp and subjected to end-repair, A-tailing, and ligation of Illumina-compatible indexed sequencing adapters.

Exonic regions were captured using the Agilent SureSelect Human All Exon capture reagent (Agilent Technologies, Santa Clara, CA, USA), targeting approximately 37–50 Mb of coding sequence and flanking intronic regions in accordance with the manufacturer’s instructions [20]. Hybrid selection was performed with biotinylated RNA baits followed by streptavidin bead capture. Post-capture libraries were amplified by PCR, purified, and quantified by qPCR and Bioanalyzer prior to sequencing. Matched tumour, preinvasive, and normal DNA from the same patient were processed in parallel to minimise batch effects.

### 2.4. Whole-Exome Sequencing

Captured libraries were sequenced on the Illumina HiSeq platform (Illumina, San Diego, CA, USA) using paired-end 2 × 100 bp reads following the manufacturer’s protocol, consistent with the sequencing workflow used in large-scale lung adenocarcinoma exome studies at the Broad Institute [4]. Basic image analysis, base calling, demultiplexing, and FASTQ generation were performed using the Illumina Real-Time Analysis (RTA) and bcl2fastq (v2.17) pipelines. Initial alignment and sequence-level quality control were carried out with the Broad Institute Picard and Firehose pipelines [4]. Per-sample sequencing quality was monitored using Picard HsMetrics; target-coverage and library-quality values for all 33 samples are provided in Supplementary Table S1.

### 2.5. Read Processing and Alignment

Raw FASTQ files were quality-assessed using FastQC (v0.11.2). Low-quality bases and residual adapter sequences were trimmed using the Sickle program (v1.33) and Trimmomatic (v0.30). Cleaned reads were aligned to the human reference genome GRCh37/hg19 using BWA-MEM (v0.7.15) with default parameters to generate BAM files. Aligned BAMs were sorted and merged with SAMtools (v1.1). PCR and optical duplicates were flagged using Picard MarkDuplicates (v2.17.3), and base quality score recalibration (BQSR) and local realignment around indels were performed using the Genome Analysis Toolkit (GATK, v4.1.2.0), following the best-practice processing framework adopted by the Broad Institute Firehose pipeline [4]. Per-sample Picard HsMetrics, including mean target coverage, percentage of target bases covered at ≥20×, mapping rate, duplicate rate, mean insert size, and inter-sample contamination, were monitored throughout processing and are tabulated in Supplementary Table S1. Across the 33 samples analysed in this study, the median mean target coverage was 64× (range 33–87×; interquartile range 55–70×), a median of 77% of targeted bases were covered at ≥20× (range 49–86%), the median fraction of PF reads aligned was 97% (range 91–98%), library duplication rates had a median of 17% (range 13–25%), and inter-sample contamination estimates (Picard ContEst) had a median of 0.02% (range 0.00–0.27%), all within the ranges expected for FFPE-compatible hybrid-capture exome sequencing and adequate for robust somatic variant calling at the VAF thresholds used below.

### 2.6. Somatic Variant Calling

Somatic single-nucleotide variants (SNVs) and small insertions/deletions (indels) were identified by paired tumour/preinvasive–normal comparison using algorithms calibrated for stromally contaminated cancer tissues, following the analytical strategy established in the Broad Institute lung adenocarcinoma exome study [4]. For each patient, both the preinvasive lesion and the invasive carcinoma were analysed against the matched normal sample. SNVs were called using MuTect (version 1.1.7) (Broad Institute) and cross-validated with GATK Mutect2, and indels were called using Indelocator (Broad Institute pipeline, circa 2014–2016)) and GATK Mutect2 [4]. Somatic variants were also cross-referenced using ADIscan (v4.0.1.2), which is optimised for paired sample analysis and has been applied to high-coverage exome data [21]. To provide an independent orthogonal variant call-set using the community-standard Broad workflow, the raw WES BAM files were additionally processed through the Broad Institute Firehose MuTect1/Indelocator pipeline [4] at the Broad Institute; the resulting MAF was used to cross-check driver-gene observations and to annotate selected variants against COSMIC and the Cancer Gene Census (functional-predictor annotations via PolyPhen-2, SIFT, FATHMM and MutationAssessor).

To retain high-confidence somatic calls, variants were required to meet the following criteria: (i) minimum tumour/preinvasive and normal coverage of 10 reads at the variant site; (ii) variant allele frequency (VAF) ≥ 5% in the tumour or preinvasive sample and <1% in the matched normal; (iii) at least three reads supporting the variant allele; and (iv) absence from a panel of normals constructed from the matched normal samples in this cohort to remove recurrent sequencing artifacts. Common germline polymorphisms listed in dbSNP (build 150) and gnomAD (v2.1), without supporting evidence in COSMIC, were filtered out unless annotated as pathogenic in ClinVar. Variants were annotated using ANNOVAR (1 February 2016) against RefSeq gene models and were cross-referenced against COSMIC, ClinVar, and the Cancer Gene Census databases to infer functional and clinical relevance. Only non-synonymous variants, including missense, nonsense, frameshift, non-frameshift indels, stop-gain, stop-loss, and splice-site mutations, were considered in downstream analyses.

Because all 33 libraries were prepared from formalin-fixed paraffin-embedded (FFPE) material, several layers of FFPE-artefact mitigation were applied during variant calling and filtering, given that formalin-induced cytosine deamination is known to generate spurious C > T (and complementary G > A) transitions at low VAF. Specifically: (i) every variant was called only in the paired matched-normal comparison and required absence from the patient’s own matched normal lung (VAF < 1%), which removes germline variants and any patient-specific FFPE artefacts that are equally prevalent in the normal compartment; (ii) the cohort-wide panel-of-normals filter described above suppressed recurrent FFPE-style artefacts that appear across multiple matched-normal libraries; (iii) MuTect/Mutect2 default filters that discard variants with strong strand bias, low base quality, or read-orientation bias—all enriched in FFPE-deamination artefacts—were applied; (iv) every reported variant was further required to be confirmed by an orthogonal caller (MuTect cross-validated with Mutect2, plus ADIscan; driver-gene observations additionally cross-checked against the Broad Institute Firehose MuTect1/Indelocator MAF), which substantially reduces caller-specific FFPE noise; and (v) the 5% VAF threshold removed the low-VAF tail in which FFPE artefacts are concentrated. We additionally note that the smoker-versus-never-smoker mutational-spectrum analysis was restricted to the cancer-only (A1) compartment, which by construction excludes variants shared with the matched normal and thereby reduces the contribution of FFPE-deamination C > T artefacts to that contrast. Despite these safeguards, residual FFPE deamination can never be eliminated entirely from FFPE-derived WES data, and low-VAF subclonal variants should accordingly be interpreted with caution; this caveat is also acknowledged in the Discussion.

### 2.7. Targeted Analysis of Cancer- and Microenvironment-Related Gene Sets

To focus the analysis on biologically relevant genes, two curated gene lists were assembled. The 684 cancer-related genes were compiled by intersecting (i) the routine clinical cancer-gene panels used for somatic variant reporting at our institution—themselves derived from clinical-grade lung-cancer panels and broader pan-cancer hotspot lists—with (ii) genes annotated as cancer-associated in the Catalogue of Somatic Mutations in Cancer (COSMIC, including the Cancer Gene Census, accessed on 1 January 2020 (https://cancer.sanger.ac.uk/cosmic), (iii) genes with cancer-pathogenic variants in ClinVar, accessed on 1 January 2020 (https://www.ncbi.nlm.nih.gov/clinvar), and (iv) genes catalogued in the NCBI Gene resource, accessed on 1 January 2020 (https://www.ncbi.nlm.nih.gov/gene) with explicit cancer-related annotation. By construction, the panel includes the canonical lung-cancer driver genes catalogued in TCGA LUAD and SqCC [2,3] and in the Broad Institute LUAD cohort [4]—*EGFR*, *KRAS*, *HRAS*, *TP53*, *STK11*, *KEAP1*, *PIK3CA*, *MET*, *BRAF*, *ALK*, *RB1*, *CDKN2A*, *SMAD4*, *ATM*, *U2AF1*, *RBM10*, and the other genes that recur in our oncoprint together with broader pan-cancer drivers (*BRCA1/2*, *APC*, *NF1*, *NOTCH1*, etc.). The panel does not, however, cover regulatory or non-coding regions, structural-rearrangement breakpoints, fusion-prone introns (e.g., *ALK*, *ROS1*, *RET* introns commonly involved in lung-cancer rearrangements), or repetitive elements; it also does not enable detection of arm-level copy-number alterations or HLA loss of heterozygosity. We acknowledge in the Discussion that these classes of variation—non-coding alterations, structural rearrangements, and copy-number/HLA-LOH events—may carry biology relevant to the preinvasive-to-invasive transition that the present design cannot capture. The 695 tumour microenvironment (TME)-related genes were compiled in parallel by querying NCBI Gene with the keywords “tumor” and “tumor microenvironment” restricted to Homo sapiens. Both gene lists are available from the corresponding author upon reasonable request.

All somatic variants (single-nucleotide and small indel) overlapping these gene sets—including exonic, splicing, 5′/3′ UTR, and intronic positions returned by the annotation pipeline—were extracted from the WES variant calls for evolutionary classification. Variants were then classified within each patient according to their VAF pattern across the matched normal (N), preinvasive (P), and carcinoma (C) samples into four clonal-evolutionary classes (see Results): cancer-only (A1: N ≈ P < C), shared-and-expanding (A2: N < P ≤ C), preinvasive-only (B1: N ≈ C < P), and shared-and-shrinking (B2: N < C < P). We emphasise that this VAF-pattern scheme is a bulk-tissue, single-region approximation of clonal dynamics and is not a substitute for formal clonal-evolution inference: it does not correct for tumour-cell purity or normal-cell contamination, does not estimate cancer-cell fractions (e.g., as in PyClone or PhyloWGS), does not resolve subclonal architecture beyond the four-bin scheme, and can be confounded by intra-lesion heterogeneity within a single bulk biopsy. We therefore use it only as a descriptive, hypothesis-generating framework to flag candidate early-versus-late events; the resulting evolutionary-class assignments should be interpreted accordingly. A formal subclonal reconstruction would require multi-region sampling of each lesion together with allele-specific copy-number information, neither of which was available in this study. Nonsynonymous (protein-altering) driver events highlighted in the Results were additionally annotated using ANNOVAR and cross-referenced against COSMIC and ClinVar for hotspot status. Patient-specific phylogenetic trees were inferred from VAF matrices, and hierarchical clustering of samples by mutation profiles was performed for visualisation; these visualisations should likewise be regarded as exploratory rather than as inferred clonal phylogenies.

### 2.8. Pathway Analysis and Functional Annotation

The cancer-related genes that were mutated exclusively in carcinoma samples (cancer-only compartment) were subjected to functional enrichment analysis with DAVID Bioinformatics Resources v6.8, accessed on 1 January 2020 (https://ngdc.cncb.ac.cn/databasecommons/database/id/3061) [22], using the Genetic Association Database (GAD) disease-class annotation. The pathogenic significance of individual driver-gene variants was reviewed in NCBI ClinVar and COSMIC.

### 2.9. Mutational Spectrum and Cancer Hallmarks Mapping

To characterise mutagenic processes, we tabulated the base-substitution spectrum of all single-nucleotide variants in the cancer-related gene set, collapsed onto the pyrimidine strand (C > A, C > G, C > T, T > A, T > C, T > G). The smoking-versus-never-smoker comparison was restricted to the cancer-only (A1) compartment, which represents variants newly acquired in the carcinoma and most directly reflects the mutagenic processes operative at the preinvasive-to-invasive transition. Differences in substitution-class frequencies between smokers (current/ex-smokers, n=6) and never-smokers (n=5) were tested by Fisher’s exact test (C > A vs. all other substitutions). For driver-gene contrasts between the pre-invasive (B1/A2) and carcinoma (A1/A2) compartments, two-sided Fisher’s exact tests were used at the patient level (11 vs. 11); within-patient paired contrasts were additionally assessed with McNemar’s exact test on discordant pairs. For cross-cohort comparison with the published 197-specimen AIS/MIA–LUAD cohort [12], per-compartment *TP53* mutation frequencies were computed separately within each cohort and compared by one-sided Fisher’s exact test (invasive > preinvasive); 95% confidence intervals were Wilson score intervals. All statistical tests were two-sided; p<0.05 was used as the threshold for statistical significance, without multiple-comparison correction given the hypothesis-generating nature of the gene-level contrasts. To evaluate the distribution of somatic alterations across core tumour-biology axes, we mapped mutated cancer-related genes in each patient to the hallmarks of cancer as operationalised in a landmark lung adenocarcinoma mapping [4], including the proposed 11th hallmark of epigenetic/RNA deregulation. Gene–hallmark assignments followed the Hanahan and Weinberg framework (e.g., *EGFR*, *KRAS*, *HRAS*, *MET*, *MYC*, *PIK3CA* → sustaining proliferative signaling; *TP53*, *RB1*, *CDKN2A*, *STK11*, *SMAD4* → evading growth suppressors; *TP53*, *ATM*, *BRCA2*, *CHEK2*, *MSH6* → genome instability; *U2AF1*, *ARID1A*, *SMARCA4* → epigenetic/RNA deregulation).

## 3. Results

### 3.1. Clinicopathologic Features of the Study Cohort

**Figure 1:**
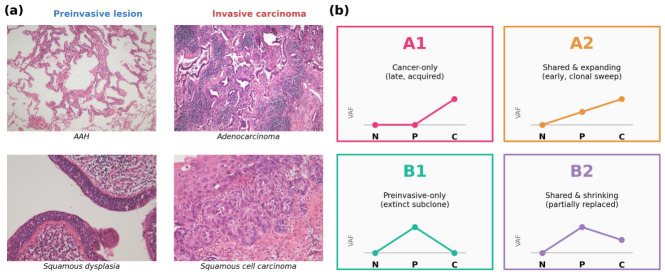
Study design and evolutionary classification framework. (**a**) Representative H&E sections of matched AAH–LUAD (**top**) and SD–SqCC (**bottom**) pairs. (**b**) Four evolutionary classes by VAF across normal (N), preinvasive (P), and carcinoma (C): A1, cancer-only (late); A2, shared/expanding (early); B1, preinvasive-only (extinct); B2, shared/shrinking.

We analysed whole-exome sequencing (WES) data from 33 samples obtained from 11 patients, comprising 7 synchronous AAH–LUAD pairs (with matched normal lung) and 4 synchronous SD–SqCC pairs (with matched normal lung). The overall study design and the evolutionary-class classification framework used throughout this work are summarised in Figure 1. Clinicopathologic characteristics of the 11 patients are presented in Table 1. The median age at surgery was 67 years (range: 51–81), and 10 of 11 patients (91%) were male. Five patients were never-smokers (four in the LUAD group: K004, K011, K016, K020; and one in the SqCC group: K003), and six were current or ex-smokers, evenly distributed between the two histologic subtypes (three in LUAD: K009, K010, K021; and three in SqCC: K006, K018, K019). This composition is consistent with the known smoking-related aetiology of SqCC and the high prevalence of EGFR-driven, never-smoker LUAD in East Asian populations. All tumours were resected at an early pathologic stage (T1a/T1b/T2a, N0–N2, M0).

### 3.2. Sequencing Quality Control of the Preinvasive–Invasive WES Cohort

All 33 libraries met accepted standards for hybrid-capture exome sequencing (median mean target coverage 64×; median 77% of target covered at ≥20×; median 97% PF reads aligned to GRCh37/hg19; inter-sample contamination ≤0.27%). Paired preinvasive, invasive, and matched normal samples within each patient were sequenced at comparable depth, supporting the VAF-based evolutionary-class assignments used below (full per-sample Picard HsMetrics in Supplementary Table S1).

### 3.3. Mutational Burden in Preinvasive and Malignant Lesions

**Figure 2:**
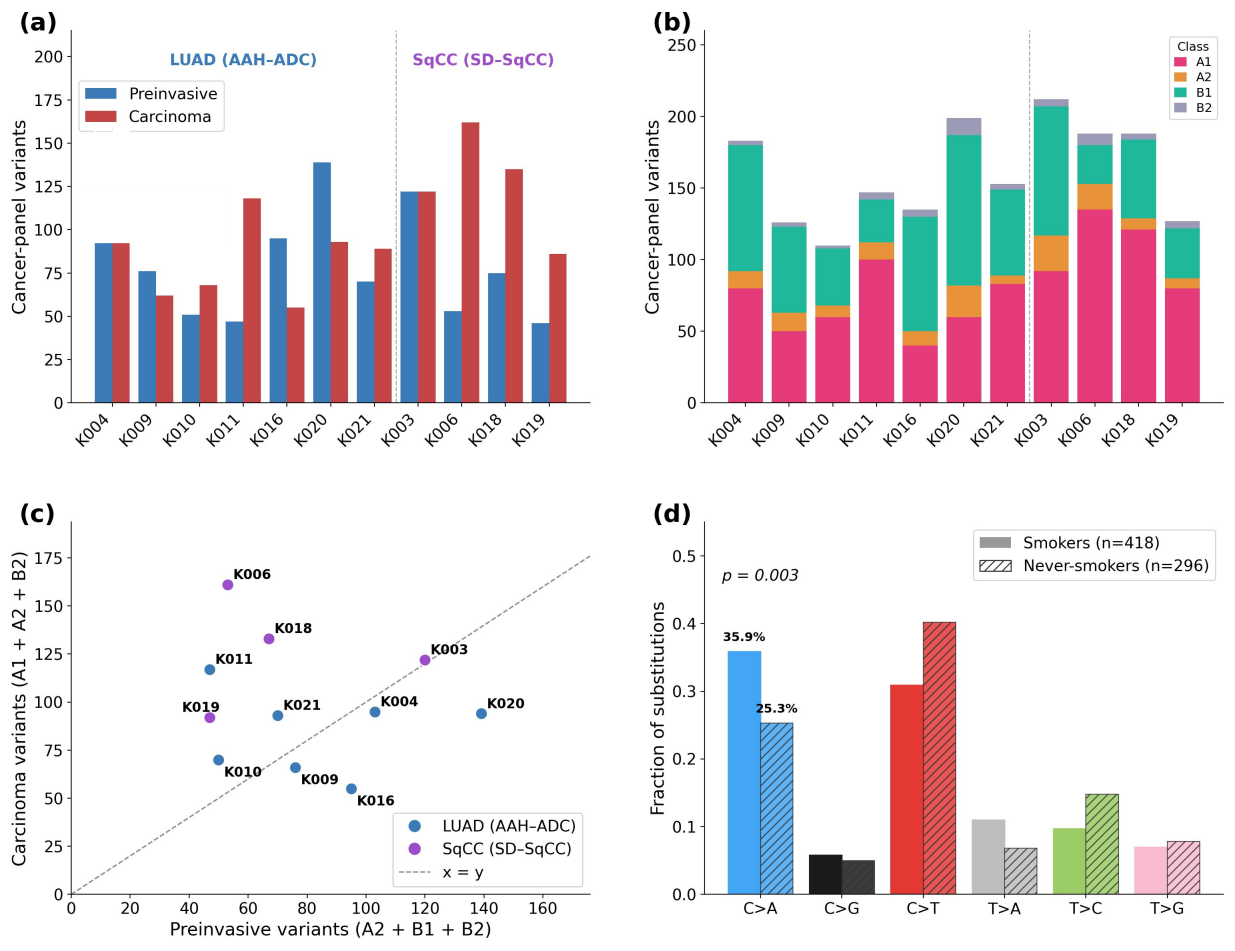
Variant burden and evolutionary-class landscape of the 11-patient cohort. (**a**) Cancer-panel variant counts in preinvasive (blue) and carcinoma (red) per patient, grouped by histology. (**b**) Per-patient stacked distribution across the four evolutionary classes (A1 cancer-only, A2 shared/expanding, B1 preinvasive-only, B2 shared/shrinking). (**c**) Preinvasive (A2 + B1 + B2) vs. carcinoma (A1 + A2 + B2) variant counts; dashed line, x=y; LUAD pairs blue, SqCC pairs purple. (**d**) Base-substitution spectrum of cancer-only (A1) variants by smoking status.

We first evaluated the overall somatic mutation burden in each sample. Nonsynonymous variants included nonsynonymous SNVs, nonframeshift substitutions, frameshift substitutions, stop-gain, stop-loss, and splice-site alterations. Consistent with the prevailing model of stepwise accumulation of somatic mutations during carcinogenesis [14,23], the number of nonsynonymous mutations tended to be higher in carcinomas than in matched preinvasive lesions in the majority of patients, although this trend was not universal: in one AAH–ADC pair (K009), the preinvasive lesion carried a comparable mutation count to the paired carcinoma (Figure 2a,c), and in K011, the carcinoma showed a substantially higher overall variant count yet shared only a minority of variants with the paired AAH, with most preinvasive variants absent from the carcinoma (large B1 fraction; Figure 2b). Both patterns are compatible with the finding that preinvasive lesions can harbour considerable mutational diversity, a subset of which is lost during subsequent clonal selection [14].

To focus on biologically relevant alterations, we intersected all somatic variant calls (SNVs and small indels) with a curated panel of 684 cancer-related genes and 695 tumour microenvironment (TME)-related genes compiled from ClinVar, COSMIC, and NCBI Gene. Across the cohort, 1780 cancer-panel variants were identified and classified as follows: 50.7% (*n* = 903) cancer-only (A1), 7.1% (*n* = 126) shared and expanding (A2), 39.0% (*n* = 694) preinvasive-only (B1), and 3.2% (*n* = 57) shared and shrinking (B2) (Figure 2b). The base-substitution spectrum of A1 variants showed a significant enrichment of C > A transversions in smokers compared with never-smokers (Fisher’s exact p=0.003; Figure 2d), consistent with the tobacco-exposure mutational signature SBS4.

### 3.4. Driver Gene Mutations Associated with Early and Late Events

**Figure 3:**
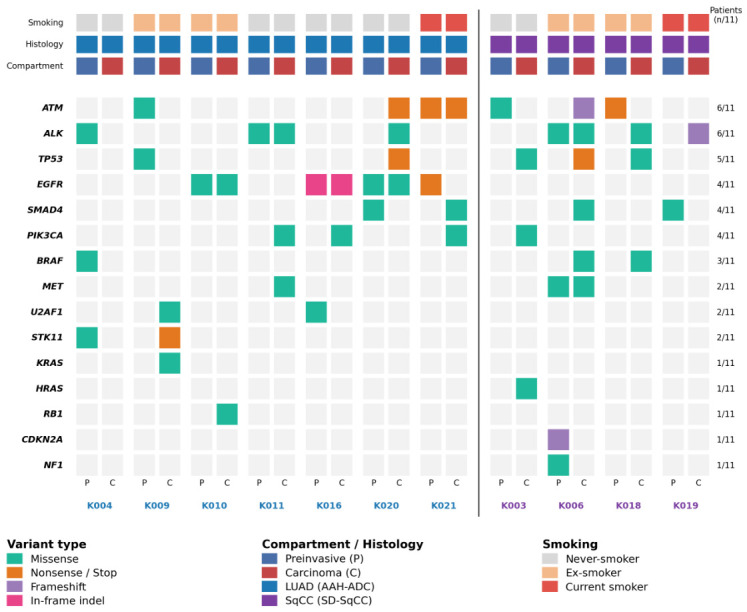
Oncoprint of driver gene alterations across the 11 synchronous preinvasive–invasive pairs. Each patient occupies two columns (P, preinvasive; C, carcinoma); LUAD on the left, SqCC on the right of the vertical divider. Cell colour denotes variant type (green, missense; orange, nonsense/stop-gain; purple, frameshift; magenta, in-frame indel). Top annotation tracks encode compartment, histology, and smoking status. Rows are 15 canonical driver genes ordered by descending number of affected patients (n/11, right). Genes coloured only in C (e.g., *TP53*, *KRAS*, *STK11*, *U2AF1* p.S34F) are late, cancer-only events; genes coloured in both P and C (e.g., *EGFR* in K010, K016) are shared early events.

**Figure 4:**
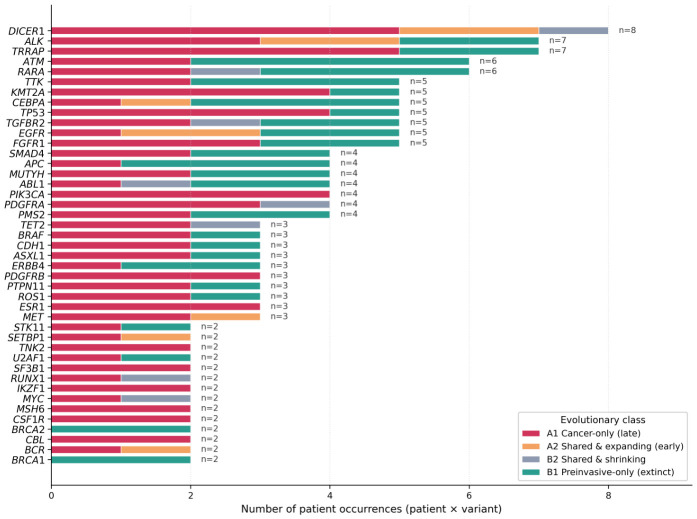
Gene-level alteration counts stratified by evolutionary class. Each bar represents one recurrently mutated cancer-related gene (≥2 affected patients); bar length is the number of patient occurrences, stacked by class: A1 (red), A2 (orange), B1 (green), B2 (grey-blue).

The predominance of preinvasive-only (B1, 39.0%) and cancer-only (A1, 50.7%) variants indicates that a substantial fraction of the genomic diversity present in AAH or squamous dysplasia is not carried forward into the paired carcinoma, consistent with a selective-sweep model of clonal evolution as reported in synchronous AAH–LUAD pairs [13], gastric adenoma–carcinoma pairs [23], and colorectal adenoma–carcinoma pairs. Focusing on protein-altering mutations in canonical driver genes, the distribution of alterations across evolutionary classes is summarised in Table 2, visualised as an oncoprint in Figure 3, and tabulated at the cohort level in Figure 4.

*TP53* was the most recurrently mutated tumour suppressor (5 of 11 patients, 45%), with 4 of 5 *TP53* mutations in the cancer-only (A1) compartment and only one (K010, p.G266V) in the preinvasive-only (B1) compartment. The per-compartment *TP53* frequency in our cohort (9% in preinvasive vs. 36% in carcinoma) closely matched an independent Chinese cohort (6% vs. 38%) [12]; the formal cross-cohort comparison is presented in Section 3.12.

In contrast, *EGFR* alterations showed a mixed temporal pattern across the four *EGFR*-mutant patients: two cases (K010, K016) harboured shared and expanding (A2) variants with comparable VAF in both AAH and LUAD, indicating clonal expansion of *EGFR*-mutant cells already present in the precursor lesion, while patient K020 carried two independent *EGFR*-activating variants in opposite temporal compartments (A1 p.L858R and B1 p.G719A). The latter pattern—two distinct activating mutations in the same driver gene within one patient—parallels observations in multiple synchronous lung cancers [24] and indicates that *EGFR* mutations appear early in glandular neoplasia and that parallel activated clones can emerge within the same host before differential selection during progression.

Other canonical drivers were almost uniformly confined to the cancer-only (A1) compartment: *KRAS* p.G12C (K009), *HRAS* p.Q61L (K003), *PIK3CA*, *STK11* p.E256 *, *SMAD4*, and *RB1* p.K65N (Table 2). Of particular interest, we identified the *U2AF1* p.S34F hotspot in the carcinoma of patient K009—a splicing-factor mutation originally reported in myelodysplastic syndromes and highlighted as a recurrent LUAD driver in the Broad Institute cohort [4]. Patient K006 (SqCC) harboured three independent *MET* variants, including a striking A2 p.E760G event whose VAF rose from 0.46 in SD to 0.89 in SqCC, indicating dominant outgrowth of one *MET*-activated subclone during malignant transition.

The full driver landscape across the 11 paired preinvasive–invasive sample columns is visualised in the oncoprint shown in Figure 3, which highlights the juxtaposition of shared, preinvasive-only, and cancer-only events in each patient. Figure 4 complements this view by summarising, for every recurrently mutated gene, the cohort-level distribution of evolutionary classes: *TP53*, *PIK3CA*, *KRAS*, *HRAS*, *RB1*, and the *U2AF1* p.S34F hotspot are dominated by A1 events, whereas *EGFR*, *ATM*, and *ALK* show a mixed A1/A2/B1 pattern consistent with a combination of early and late driver events.

### 3.5. Functional Enrichment of Cancer-Specific Mutations

DAVID Bioinformatics pathway analysis [22] of the 48 cancer-related genes mutated exclusively in the carcinoma compartment across the 11 patients showed significant enrichment in cancer, metabolism, and immune-response categories. The immune-category enrichment in the cancer-specific compartment is concordant with previous work in AAH progression, demonstrating early immune dysregulation and upregulation of immune checkpoints such as CTLA-4 during the AAH-to-LUAD transition [11], with airway-field transcriptomic evidence of immune-metabolic reprogramming in bronchial premalignancy [6].

### 3.6. Mutation Spectrum and Smoking-Associated Signatures

As shown in Figure 2d, the cancer-only (A1) compartment showed the expected smoker/never-smoker dichotomy: C > A transversions were enriched in smokers (consistent with tobacco SBS4), while C > T transitions—attributable to age-related CpG deamination—predominated in never-smokers (40.2% vs. 30.9%). This recapitulates the mutational-signature stratification reported for 183 LUADs in the Broad Institute cohort [4] and the tobacco-signature enrichment reported in smoker-derived preinvasive lesions [13]. Deeper signature deconvolution (e.g., COSMIC SBS4, SBS2/13) was not attempted because the per-lesion SNV count was below the ∼100-variant threshold recommended for stable de novo extraction.

Within-patient comparison of the A1 (cancer-only) and B1 (preinvasive-only) compartments revealed a statistically significant difference in mutagenic processes in one patient and borderline trends in two further patients. In never-smoker K016, C > A accounted for 47% of preinvasive-only substitutions but only 10% of cancer-only substitutions (Fisher’s exact $p=5\times 10^{-4}$). In ex-smoker K006, the pattern was reversed (36% cancer-only vs. 59% preinvasive-only, p=0.057), and patient K003 showed a third shift in the same direction (p=0.059); both should be considered suggestive rather than definitive and require validation in larger cohorts. Similar between-lesion spectrum discordances have been reported in multiple synchronous lung cancers [24], and our observation—most clearly in K016—extends this to the preinvasive-versus-invasive contrast within the same lesion focus, suggesting that the mutational processes operating in AAH or SD may differ from those generating the adjacent carcinoma even on an identical germline background.

### 3.7. Driver Gene Co-Occurrence and Cases Without Detected Oncogenic Drivers

The most notable co-occurrence in our cohort was activating *EGFR* with inactivating *TP53* in the carcinoma compartment of patients K010 and K020 (2/11, 18%), a pairing also reported in AAH/adenocarcinoma pairs [14] and consistent with the model of *EGFR* as an early initiator and *TP53* loss as a late event that accompanies invasion. Individual patients also showed complex multi-pathway disruption (e.g., *KRAS*–*STK11*–*U2AF1* in K009; *TP53*–*SMAD4*–*CDKN2A*–*ATM*–*MET* in K006).

An orthogonal Broad Institute MuTect/Firehose reanalysis of the raw WES BAMs identified protein-truncating *RBM10* alterations in 3/11 patients (K004, K010, K021; 27%), consistent with the frequency reported in AIS/MIA [12]. Notably, *RBM10* alterations in K010 and K021 were shared between AAH and the paired LUAD (e.g., K010 p.Q245 * VAF 0.56→0.47), indicating that *RBM10* loss-of-function can occur as an early event in AAH–LUAD evolution.

Conversely, 3 of 11 patients (K004, K011, K019; 27%) harboured no mutation in any canonical lung cancer driver gene despite substantial cancer-panel variant burdens (126–183 variants). This parallels the “driver-negative” subset reported in both the Broad LUAD cohort [4] and an AAH cohort [13], and is an important finding because it implies that a meaningful fraction of preinvasive–invasive transitions may not be explained by point mutations in canonical driver genes alone. Several alternative mechanisms could plausibly underlie malignant transformation in these patients but cannot be evaluated by our WES-only design, including structural variants and gene fusions (e.g., *ALK*, *ROS1*, *RET* rearrangements with intronic breakpoints outside our panel), arm-level copy-number alterations and HLA-LOH (identified as major preinvasive-to-invasive axes by Chen et al. [12]), non-coding regulatory variants, and epigenetic events such as promoter hypermethylation. These three patients should therefore be regarded as a flagged subset warranting follow-up with copy-number-aware, structural-variant-aware, and methylation-aware platforms.

### 3.8. Mapping Genomic Alterations to the Hallmarks of Cancer

**Figure 5:**
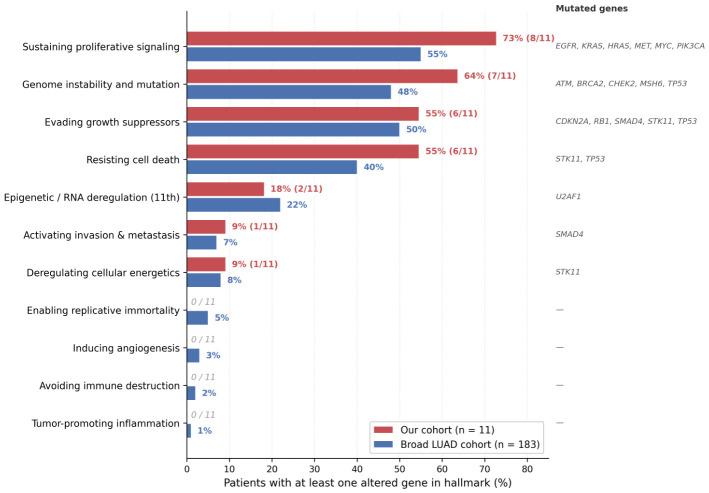
Hallmarks of cancer mapped to the 11-patient cohort versus the Broad Institute LUAD cohort (n=183). Red bars, our cohort; blue bars, Broad LUAD cohort. Rows follow the Hanahan–Weinberg framework; contributing genes in our cohort are listed at right.

Following the Hanahan–Weinberg framework [25] as operationalised in a landmark lung adenocarcinoma mapping [4], we mapped the mutated genes in each patient to the core cancer hallmarks and compared hallmark prevalence against the 183-patient Broad Institute LUAD cohort (Figure 5). Sustaining proliferative signaling was the most frequently altered hallmark in our cohort (73% vs. 55% in the Broad cohort), likely reflecting the East Asian composition with a higher baseline *EGFR*/*KRAS* mutation frequency. Consistent with the proposed 11th hallmark of epigenetic/RNA deregulation [4], 2 of 11 patients (both LUAD) harboured *U2AF1* mutations, including the MDS-associated p.S34F hotspot in K009. The hallmarks of replicative immortality, angiogenesis, immune evasion, and tumour-promoting inflammation were not attributable to any recurrently mutated gene in our cohort, echoing the finding that these hallmarks remain poorly explained by point mutations alone.

### 3.9. Intrapatient Heterogeneity and Clonal Relationships

The oncoprint (Figure 3) and per-patient evolutionary-class distribution (Figure 2b) together recapitulate a pattern of early divergence followed by parallel evolution: in most patients, the preinvasive and invasive lesions share a common trunk of early drivers (e.g., *EGFR* p.L858R in K010) while lesion-specific branches contain either late cancer-only events (*TP53*, *KRAS*, *PIK3CA*, *U2AF1*) or preinvasive-only subclones lost during progression. This mirrors the parallel-evolution model recently described in synchronous gastric adenoma–carcinoma pairs [23]. Cancer-related mutation profiles grouped samples primarily by patient rather than by histologic subtype, indicating that within-patient genomic similarity exceeds between-patient similarity of the same histology.

### 3.10. Correlations Between Clinical Features and Genomic Findings

To examine whether the genomic patterns observed above tracked with available clinical variables, we cross-tabulated the per-patient driver landscape against smoking status, histologic subtype, sex, age at surgery, and pathologic stage (Table 1 and Table 2). Three patterns are noted, recognising that with n=11, all of these are descriptive observations. First, the association between smoking status and mutational spectrum was internally consistent: all four LUAD patients with detected *EGFR* alterations were never-smokers or light/ex-smokers (K010, K016, K020 never-smoker; K021 current-smoker), whereas the single tobacco-signature-rich LUAD patient with a cancer-only *KRAS* p.G12C (K009) was an ex-smoker with 45 pack-years—the canonical never-smoker–*EGFR* versus smoker–*KRAS* dichotomy in miniature [2,4]. Second, histologic subtype tracked with the early-driver landscape (*EGFR*-shared in AAH–LUAD, *MET*-pathway-enriched in SD–SqCC) but not with the cancer-specific *TP53* signal, which was present in both LUAD and SqCC carcinomas. Third, the three driver-negative patients did not share an obvious clinical commonality, spanning both histologies, sexes, and three smoking categories; the driver-negative phenotype thus does not appear to reduce to a single clinical risk factor in this cohort. Formal statistical association and outcome correlations were not attempted at this cohort size.

### 3.11. Mutations in Tumour Microenvironment-Related Genes

In parallel with the cancer-gene analysis, we examined somatic alterations in a curated set of 695 tumour microenvironment (TME)-related genes covering extracellular matrix remodelling, cytokine and chemokine signalling, antigen presentation, immune checkpoint, and stromal–vascular regulation. Across the 11 patients, TME-gene variants showed the same patient-dominant clustering pattern as the cancer-gene set (i.e., samples grouped primarily by patient rather than by histology), and the four evolutionary-class proportions in TME genes broadly mirrored those of the cancer panel, with a substantial preinvasive-only (B1) fraction. The DAVID functional-enrichment analysis of the 48 genes mutated exclusively in the carcinoma compartment returned significant over-representation of immune-response, antigen-processing, and cytokine categories, consistent with a shift in the local immune milieu accompanying the preinvasive-to-invasive transition. Together with the known Th1/Th2 imbalance and checkpoint upregulation reported in AAH [11] and the airway field-of-injury transcriptomic signature that discriminates progressing from regressing bronchial premalignancy [6], these mutation-level observations are compatible with the broader view that the TME co-evolves with the malignant compartment. We interpret these findings as hypothesis-generating evidence supporting parallel microenvironmental evolution rather than as a complete TME characterisation, noting that the principal mechanisms by which the TME is reshaped—transcriptional reprogramming, cellular composition shifts, and HLA-LOH—are not captured by bulk-tissue WES.

### 3.12. Cross-Cohort Validation of TP53 as a Late Event in Lung Carcinogenesis

**Figure 6:**
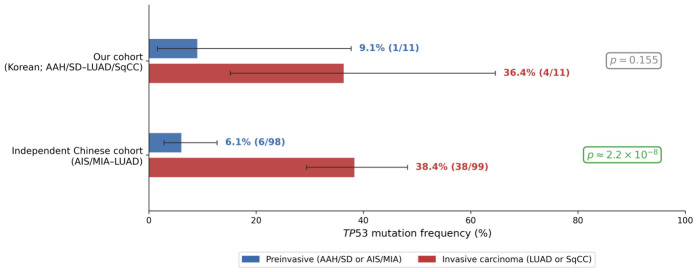
Cross-cohort *TP53* mutation frequency: preinvasive (blue) vs. invasive (red). (**Top**) Korean cohort; (**bottom**) Chinese cohort. Error bars, 95% Wilson score CIs.

To place the *TP53* late-event signal in a broader context, we compared our cohort with the independent 197-specimen Chinese AIS/MIA–LUAD cohort [12] (Figure 6). Within our cohort, *TP53* mutation frequency was numerically higher in the carcinoma than in the paired preinvasive compartment (4/11, 36.4% vs. 1/11, 9.1%), although this contrast did not reach statistical significance (p=0.155, one-sided Fisher’s exact). The Chinese AIS/MIA–LUAD cohort showed the same direction with a much stronger statistical signal (38.4% vs. 6.1%, $p=2.2\times 10^{-8}$). Notably, the per-compartment frequencies were similar across both cohorts despite our preinvasive compartment comprising benign-appearing AAH/SD rather than AIS/MIA, suggesting that the late-event *TP53* pattern observed in AIS/MIA–LUAD pairs is already detectable in benign-appearing preinvasive lesions. Given our small sample size and non-significant within-cohort *p*-value, this observation is hypothesis-generating rather than confirmatory.

## 4. Discussion

The principal observation of this work is not a single recurrent driver mutation but a structural feature of early lung carcinogenesis: in our cohort, the dominant molecular events at the preinvasive stage appear to diverge between glandular and squamous tracks, while late, cancer-specific events partially converge. In the glandular track, the early signal is a shared receptor tyrosine kinase activation event—most clearly *EGFR*—already present in the histologically benign AAH compartment. In the squamous track, no shared activating receptor tyrosine kinase event was detectable in our four SD–SqCC pairs, and the dominant early signal was pathway-level *MET* activation (with one patient, K006, contributing three of the four *MET* variants). Late, cancer-specific events—most prominently *TP53*—then accumulate on both tracks, and the same directional pattern was observed in the larger Chen et al. AIS/MIA–LUAD cohort [12]. Given the cohort size (in particular n = 4 SqCC pairs) and the non-significant within-cohort *TP53* contrast (p=0.155), all these observations are hypothesis-level. With these caveats in mind, the most striking implication of our data is biological rather than diagnostic. At the cancer-panel level, AAH and low-grade SD shared only a minority of variants with their paired carcinomas (approximately 10% shared, versus 50% cancer-only and 40% preinvasive-only), indicating that the benign-appearing compartment is not a broadly attenuated version of the paired carcinoma but a largely separable genomic entity. The signal that does cross the preinvasive–invasive boundary is concentrated in a small set of key drivers whose identity appears to depend on the histologic lineage—most clearly shared *EGFR* alterations in AAH–LUAD pairs and *MET*-pathway events in SD–SqCC pairs—rather than in a broad cancer-committed program. By extension, this argues that benign-appearing AAH/SD are likely distinguishable at the genomic as well as the histologic level from the more advanced AIS/MIA/CIS precursors profiled in dedicated cohorts [8,12,13], supporting the view that the benign and in situ/minimally invasive tiers of lung preinvasive disease are not biologically interchangeable. These observations are consistent with—but, given the small cohort, do not by themselves establish—a lineage-specific and classification-tier-specific framework for early lung neoplasia.

These findings extend, rather than replicate, three classes of prior studies. Mutation-centric profiling of AAH/AIS/MIA cohorts established that EGFR-mutant subclones can be detected in AAH and become dominant in AIS/MIA/ADC [11,13,14]; we confirm this pattern in synchronous AAH–LUAD pairs from a Korean population. Multi-omic profiling of 98 AIS/MIA and 99 LUAD specimens identified *TP53* mutation, arm-level copy-number alterations, and HLA loss of heterozygosity as the alterations most enriched in the invasive stage [12]; the *TP53*-enrichment component was directionally concordant with our data, whereas the copy-number and HLA-LOH components could not be evaluated in our WES-only design. The multiple synchronous lung cancer cohort [24] concluded that distinct intra-thoracic lesions typically represent independent primaries with convergent driver selection; our paired preinvasive–invasive design differs in that both lesions arise from the same focus, but the within-patient mutational-spectrum discordance we observed—most clearly in K016—raises the possibility that even within a single lesion focus the AAH and LUAD compartments may not always represent a strict linear precursor–successor pair. We therefore view the present cohort as a complementary, synchronous-pair data point in the emerging map of early lung carcinogenesis.

Two practical implications follow from these biological observations. First, the substantial preinvasive-only (B1) variant fraction observed in our cohort (39% of cancer-panel variants) confirms that not every variant detectable in AAH or SD is retained in the paired carcinoma; mutation-only panels applied to preinvasive biopsies will therefore yield false-positive risk signals unless interpreted alongside the evolutionary class of each variant. Second, because the early-driver landscape differs between histologic tracks, any risk-stratification strategy for benign-appearing lung precursors should be histology-aware: an EGFR-positive AAH is plausibly a higher-risk lesion warranting closer follow-up, whereas SD lacks an equivalent single-gene marker and may require a copy-number-, methylation-, or immune-microenvironment-based biomarker. Both possibilities require prospective validation before any clinical recommendation can be made.

Several substantive limitations of this study must be acknowledged before these interpretations can be generalised. The most important is sample size: with 7 AAH–LUAD and 4 SD–SqCC pairs, our cohort is one to two orders of magnitude smaller than published reference cohorts [2,4,12,13], which precludes the identification of significantly mutated genes by standard cohort-level methods and leaves all per-gene contrasts as hypothesis-generating; the SqCC arm in particular (*n* = 4) is too small to support any general statement about the SD–SqCC track in isolation, including the *MET*-pathway pattern emphasised above. We also did not perform functional or experimental validation of any of the candidate early or late driver events identified by sequencing—no orthogonal confirmation by RNA-seq, protein-level assay, or isogenic cell-line modelling was attempted—and we did not test the predicted progression-risk implications in an independent prospective cohort: cross-cohort comparison with the Chinese AIS/MIA–LUAD cohort [12] shows a directionally consistent pattern for *TP53*, but most of our other observations (the SqCC-specific *MET*-pathway pattern, the lineage-specific divergence of early drivers, the within-patient spectrum discordance in K016) remain unvalidated. A further limitation concerns the omics scope of the study. WES alone cannot resolve allele-specific copy-number alterations, structural rearrangements and gene fusions, HLA loss of heterozygosity, transcriptomic state, or methylation, and none of these were measured here; Chen et al. identified arm-level copy-number alterations and HLA-LOH as major axes of the preinvasive-to-invasive transition [12] that our design cannot evaluate, and in the SqCC arm—where the dominant biology is recognised to be copy-number-driven—this omission means that our SqCC findings should be regarded as a partial view. The TME analysis suffers from the same constraint: the principal mechanisms of TME remodelling (cellular composition shifts, transcriptional reprogramming, immune checkpoint dynamics, HLA-LOH) are not captured by bulk-tissue WES, and a complete TME characterisation will require single-cell or spatial transcriptomics, immune profiling, and HLA-LOH calling. Two further design constraints deserve mention. Our VAF-pattern-based four-class evolutionary scheme is a descriptive, bulk-tissue approximation of clonal dynamics rather than a formal subclonal reconstruction: it does not estimate cancer-cell fractions or perform tumour-purity correction (as in PyClone or PhyloWGS), does not resolve subclonal architecture beyond the four bins, and is single-region; we therefore regard the resulting class assignments as a flagging tool for candidate early-versus-late events rather than as inferred clonal phylogenies. We also did not perform multi-region sampling of individual lesions, so we cannot formally exclude that in some patients (e.g., K020 with two distinct *EGFR* variants, K016 with the AAH/LUAD spectrum discordance) the preinvasive and invasive clones represent genetically related but multifocal primaries rather than a strict linear precursor–successor pair [26]. All 33 libraries were prepared from FFPE tissue, which is known to generate formalin-deamination C > T/G > A artefacts, particularly at low VAF; although these were mitigated by paired-normal subtraction, panel-of-normals filtering, orthogonal caller cross-validation, MuTect strand-bias and read-orientation filters, and a 5% VAF threshold, low-VAF B1 (preinvasive-only) calls should be interpreted with corresponding caution. Finally, because we captured synchronous lesions at a single time point, the inferred temporal ordering rests on VAF logic rather than direct longitudinal observation, and follow-up is too short for outcome-based association—a substantive limitation for any translational claim about risk stratification. The cohort is also drawn from a single Korean institution with imbalanced sex (10/11 male) and never-smoker-enriched LUAD, which limits generalisability to Western, smoking-driven LUAD populations; prior Korean work on small lung adenocarcinomas has likewise emphasised the central role of *EGFR* mutation and p53 dysregulation during multistage progression in this population [27].

Addressing these limitations defines a concrete agenda for follow-up work. The sample size and validation gap can only be closed by larger, prospectively collected, ethnically and clinically diverse synchronous-pair cohorts—ideally drawn from multi-institutional consortia such that both AAH–LUAD and SD–SqCC arms are independently powered. Future studies should also be multi-omic by design: layering allele-specific copy-number analysis, structural-variant and fusion calling (e.g., RNA-based fusion detection for *ALK*/*ROS1*/*RET*-like rearrangements), HLA-LOH calling (e.g., LOHHLA), bulk and single-cell RNA sequencing, and DNA methylation profiling onto the same paired-lesion specimens would directly test whether the lineage-specific early-driver pattern described here is reinforced or rewritten when copy-number, structural, transcriptional, and epigenetic axes are included, and would specifically address the driver-negative subset described above. The candidate early and late driver events—particularly the *MET*-pathway expansion in SD–SqCC pairs and the role of *TP53* as a late event in both tracks—warrant direct functional validation through orthogonal expression measurement (RNA-seq, immunohistochemistry, or proteomic profiling) and through perturbation in patient-derived organoids, isogenic cell models, or genetically engineered preclinical systems; complementary mutational analyses of *EGFR*, HER2, and *KRAS* in AAH-associated foci have similarly demonstrated that a genotypic relationship between AAH and its paired adenocarcinoma can be formally established with targeted assays [28]. To convert these descriptive observations into translationally meaningful risk stratification, future synchronous-pair studies should be linked from the outset to prospectively annotated long-term clinical outcomes (recurrence, metachronous primaries, overall survival), so that genomic features can be formally tested as predictors rather than inferred descriptively. Longitudinal sampling, either through serial bronchoscopic biopsy of bronchial dysplasia or through plasma/sputum liquid-biopsy detection of preinvasive-only variants [7,14], will be required to convert the inferred temporal ordering into directly observed clonal dynamics and, ultimately, to identify the molecular features that distinguish progressing from regressing benign-appearing lesions in real time. Together, these directions should transition early-lung-neoplasia molecular profiling from the descriptive, hypothesis-generating stage represented by the present work to a validated, clinically actionable risk-stratification framework.

## 5. Conclusions

In this paired analysis of synchronous preinvasive and invasive lung lesions from 11 patients, histologically benign-appearing AAH and low-grade SD were genomically largely distinct from their paired carcinomas—sharing only a minority of cancer-panel variants across the preinvasive–invasive boundary—while harbouring a small set of identifiable key drivers whose dominant early signal appeared to differ between the two histologic lineages: shared receptor–tyrosine–kinase activation (*EGFR*) was the prominent early event in our AAH–LUAD pairs, whereas the four SD–SqCC pairs showed no shared *EGFR* events and instead featured early *MET*-pathway activation. *TP53* inactivation appeared as a late, invasion-associated event in both lineages; we emphasise that within our 11-patient cohort, the preinvasive-versus-carcinoma *TP53* contrast was direction-consistent but did not reach statistical significance, and that a comparable directional pattern was observed in the independent Chen et al. AIS/MIA–LUAD cohort [12], rendering this observation hypothesis-generating rather than confirmatory. Together, these findings argue that benign-appearing AAH/SD are not simply less-advanced AIS/MIA but a separable category of preinvasive disease in both histologic and genomic terms.

Given the small sample size and the absence of functional, copy-number, transcriptomic, and outcome data, these findings should be regarded as preliminary and hypothesis-generating; the pathway-specific (*EGFR*-versus *MET*-)driven divergence in particular requires confirmation in independently powered AAH–LUAD and SD–SqCC cohorts. They do, however, provide a structured set of testable predictions for the next generation of larger, multi-omic, longitudinal, outcome-linked studies of early lung neoplasia, complementing earlier commentary on the value of premalignant genomic data for reconstructing the evolutionary trajectory of lung adenocarcinoma [29] and broader surveys of the *EGFR* mutational landscape in LUAD [30].

## Figures and Tables

**Table 1:** Clinicopathologic characteristics of the 11 patients included in the study.

Patient	Group	Age	Sex	Smoking (Pack-Years)	Stage	Preinvasive	Invasive (Pattern)
K004	LUAD	67	F	Never	T2aN0M0	AAH	ADC (acinar)
K009	LUAD	65	M	Ex (45)	T1bN0M0	AAH	ADC (papillary)
K010	LUAD	73	M	Ex (30)	T1bN0M0	AAH	ADC (solid)
K011	LUAD	55	M	Never	T2aN0M0	AAH	ADC (acinar)
K016	LUAD	58	M	Never	T1aN0M0	AAH	ADC (acinar)
K020	LUAD	51	M	Never	T1aN0M0	AAH	ADC (acinar)
K021	LUAD	74	M	Current (50)	T2aN0M0	AAH	ADC (acinar)
K003	SqCC	62	M	Never	T1aN0M0	SD, mild	SqCC
K006	SqCC	75	M	Ex (40)	T1aN0M0	SD, mild	SqCC
K018	SqCC	72	M	Ex (45)	T1aN0M0	SD, moderate	SqCC
K019	SqCC	81	M	Current (70)	T1aN0M0	SD, mild	SqCC

AAH, atypical adenomatous hyperplasia; ADC, adenocarcinoma; LUAD, lung adenocarcinoma; SD, squamous dysplasia; SqCC, squamous cell carcinoma.

**Table 2:** Driver gene alterations identified in the 11-patient cohort.

Gene	Patient	Histology	Protein Change	Class	VAF (P → C)	COSMIC ID	Tier
*TP53*	K020	LUAD	p.Q61 *	A1	0.00 → 0.42	COSM44032	IV
*TP53*	K010	LUAD	p.G266V	B1	0.31 → 0.00	COSM99952	IV
*TP53*	K003	SqCC	p.R26L	A1	0.00 → 0.38	COSM10714	IV
*TP53*	K006	SqCC	p.Q60 *	A1	0.00 → 0.11	COSM117949	IV
*TP53*	K018	SqCC	p.Y234C	A1	0.00 → 0.27	COSM3388193	IV
*EGFR*	K020	LUAD	p.L858R	A1	0.00 → 0.30	COSM6224	I-A
*EGFR*	K010	LUAD	p.L858R	A2	0.23 → 0.26	COSM6224	I-A
*EGFR*	K016	LUAD	exon 20 ins (p.773insVH)	A2	0.10 → 0.11	COSM404810	I-A
*EGFR*	K020	LUAD	p.G719A	B1	0.19 → 0.00	COSM6239	I-A
*EGFR*	K021	LUAD	p.E134 *	B1	0.05 → 0.00	—	IV
*KRAS*	K009	LUAD	p.G12C	A1	0.00 → 0.21	COSM516	I-A
*HRAS*	K003	SqCC	p.Q61L	A1	0.00 → 0.13	COSM498	III
*PIK3CA*	K016	LUAD	p.M1043V	A1	0.00 → 0.20	COSM94983	II
*PIK3CA*	K021	LUAD	p.E707K	A1	0.00 → 0.17	COSM5030972	III
*PIK3CA*	K011	LUAD	p.S509Y	A1	0.00 → 0.07	—	III
*PIK3CA*	K003	SqCC	p.V73I	A1	0.00 → 0.07	—	IV
*STK11*	K009	LUAD	p.E256 *	A1	0.00 → 0.05	COSM5731897	II
*STK11*	K004	LUAD	p.G227V	B1	0.06 → 0.00	—	IV
*U2AF1*	K009	LUAD	p.S34F	A1	0.00 → 0.43	COSM166866	II
*U2AF1*	K016	LUAD	p.S231L	B1	0.06 → 0.00	COSM1130534	IV
*SMAD4*	K021	LUAD	p.V437F	A1	0.00 → 0.06	—	IV
*SMAD4*	K020	LUAD	p.V437F	B1	0.06 → 0.00	—	IV
*SMAD4*	K006	SqCC	p.R380S	A1	0.00 → 0.07	COSM6913975	III
*SMAD4*	K019	SqCC	p.V437F	B1	0.07 → 0.00	—	IV
*MET*	K011	LUAD	p.T301K	A1	0.00 → 0.07	—	III
*MET*	K006	SqCC	p.E760G	A2	0.46 → 0.89	COSM6191595	II
*MET*	K006	SqCC	p.E302K	A1	0.00 → 0.06	—	III
*MET*	K006	SqCC	p.V465F	A1	0.00 → 0.06	—	III
*RB1*	K010	LUAD	p.K65N	A1	0.00 → 0.09	COSM284490	III
*CDKN2A*	K006	SqCC	frameshift (indel)	B1	0.05 → 0.00	COSM3092239	II
*NF1*	K006	SqCC	p.E1384K	B1	0.20 → 0.00	—	III

VAF, variant allele frequency; P, preinvasive; C, carcinoma. OncoKB/ESCAT tier: I-A, approved drug in indication; II, off-label clinical evidence; III, preclinical evidence; IV, hypothetical/passenger. *, stop codon.

## Data Availability

The data presented in this study are available on request from the corresponding author due to ethical and institutional restrictions on patient-derived genomic data. Summary variant tables and sequencing quality-control metrics that support the findings of this study are included within the article and its Supplementary Materials. Raw whole-exome sequencing data may be shared subject to Institutional Review Board approval and applicable data-sharing regulations.

## Supplementary Materials

The following supporting information can be downloaded at: https://www.mdpi.com/article/10.3390/cancers18111691/s1, Table S1: Whole-exome sequencing quality-control metrics (Picard HsMetrics) for all 33 samples (11 preinvasive, 11 invasive, 11 matched normal) analysed in this study.

## Abbreviations

The following abbreviations are used in this manuscript:

AAH	Atypical adenomatous hyperplasia
ADC	Adenocarcinoma
AIS	Adenocarcinoma in situ
CIS	Carcinoma in situ
COSMIC	Catalogue Of Somatic Mutations In Cancer
GAD	Genetic Association Database
LUAD	Lung adenocarcinoma
MIA	Minimally invasive adenocarcinoma
NSCLC	Non–small cell lung cancer
SD	Squamous dysplasia
SNV	Single nucleotide variant
SqCC	Squamous cell carcinoma
TME	Tumour microenvironment
VAF	Variant allele frequency
WES	Whole-exome sequencing

## References

[1] Jemal A., Bray F., Center M.M., Ferlay J., Ward E., Forman D. (2011). Global cancer statistics. CA Cancer J. Clin..

[2] Cancer Genome Atlas Research Network (2014). Comprehensive molecular profiling of lung adenocarcinoma. Nature.

[3] Cancer Genome Atlas Research Network (2012). Comprehensive genomic characterization of squamous cell lung cancers. Nature.

[4] Imielinski M., Berger A.H., Hammerman P.S., Hernandez B., Pugh T.J., Hodis E., Cho J., Suh J., Capelletti M., Sivachenko A. (2012). Mapping the hallmarks of lung adenocarcinoma with massively parallel sequencing. Cell.

[5] Mori M., Rao S.K., Popper H.H., Cagle P.T., Fraire A.E. (2001). Atypical adenomatous hyperplasia of the lung: A probable forerunner in the development of adenocarcinoma of the lung. Mod. Pathol..

[6] Beane J.E., Mazzilli S.A., Campbell J.D., Duclos G., Iamshchikov V., Sagers J., Anderlind C., Perdomo C., Getts M., Burman M. (2019). Molecular subtyping reveals immune alterations associated with progression of bronchial premalignant lesions. Nat. Commun..

[7] Spiro S.G., Shah P.L., Rintoul R.C., George J., Janes S., Callister M., Novelli M., Shaw P., Kocjan G., Griffiths C. (2019). Sequential screening for lung cancer in a high-risk group: Randomised controlled trial. Eur. Respir. J..

[8] Yambayev I., Sullivan T.B., Suzuki K., Zhao Q., Higgins S.E., Yilmaz O.H., Litle V.R., Moreira P., Servais E.L., Stock C.T. (2021). Pulmonary adenocarcinomas of low malignant potential: Proposed criteria to expand the spectrum beyond adenocarcinoma in situ and minimally invasive adenocarcinoma. Am. J. Surg. Pathol..

[9] Breuer R.H., Pasic A., Smit E.F., van Vliet E., Vonk Noordegraaf A., Risse E.J., Postmus P.E., Sutedja T.G. (2005). The natural course of preneoplastic lesions in bronchial epithelium. Clin. Cancer Res..

[10] Wistuba I.I., Gazdar A.F. (2006). Lung cancer preneoplasia. Annu. Rev. Pathol..

[11] Sivakumar S., San Lucas F.A., McDowell T.L., Lang W., Xu L., Fujimoto J., Zhang J., Futreal P.A., Fukuoka J., Yatabe Y. (2017). Genomic landscape of atypical adenomatous hyperplasia reveals divergent modes to lung adenocarcinoma. Cancer Res..

[12] Chen H., Carrot-Zhang J., Zhao Y., Hu H., Freeman S.S., Yu S., Ha G., Taylor A.M., Berger A.C., Westlake L. (2019). Genomic and immune profiling of pre-invasive lung adenocarcinoma. Nat. Commun..

[13] Hu X., Fujimoto J., Ying L., Fukuoka J., Ashizawa K., Sun W., Reuben A., Chow C.-W., McGranahan N., Chen R. (2019). Multi-region exome sequencing reveals genomic evolution from preneoplasia to lung adenocarcinoma. Nat. Commun..

[14] Izumchenko E., Chang X., Brait M., Fertig E., Kagohara L.T., Bedi A., Marchionni L., Agrawal N., Ravi R., Jones S. (2015). Targeted sequencing reveals clonal genetic changes in the progression of early lung neoplasms and paired circulating DNA. Nat. Commun..

[15] Mino-Kenudson M., Berezowska S., Minami Y., Chen S., Ray M.A., Rerkpichaisuth V., Hashisako M., Losmanova T., Hayashi T., Shim H.-S. (2025). A grading system for resected invasive squamous cell carcinoma of the lung: A multi-institutional study by the IASLC Pathology Committee. J. Thorac. Oncol..

[16] Ahn S., Lim J., Park S.Y., Kim H., Kwon H.J., Han Y.B., Lee C.-T., Cho S., Chung J.-H. (2020). Genetic alterations in preinvasive lung synchronous lesions. Cancer Res. Treat..

[17] Nachmanson D., Steward J., Yao H., Officer A., Jeong E., O’Keefe T.J., Hasteh F., Jepsen K., Hirst G.L., Esserman L.J. (2020). Mutational profiling of micro-dissected pre-malignant lesions from archived specimens. BMC Med. Genom..

[18] Covaris, Inc. (2020). truXTRAC^®^ FFPE DNA Kit: Application Note M020015.

[19] Zhu Q., Hu Q., Shepherd L., Wang J., Wei L., Morrison C.D., Conroy J.M., Glenn S.T., Davis W., Kwan M.L. (2015). The impact of DNA input amount and DNA source on the performance of whole-exome sequencing in cancer epidemiology. Cancer Epidemiol. Biomark. Prev..

[20] Fisher S., Barry A., Abreu J., Minie B., Nolan J., Delorey T.M., Young G., Fennell T.J., Allen A., Ambrogio L. (2011). A scalable, fully automated process for construction of sequence-ready human exome targeted capture libraries. Genome Biol..

[21] Cho Y., Lee S., Hong J., Kim B., Hong W., Jung J., Lee H.-B., Sung J., Kim H.-N., Kim H.-L. (2018). Development of the variant calling algorithm, ADIScan, and its use to estimate discordant sequences between monozygotic twins. Nucleic Acids Res..

[22] Huang D.W., Sherman B.T., Lempicki R.A. (2009). Systematic and integrative analysis of large gene lists using DAVID bioinformatics resources. Nat. Protoc..

[23] Jung S.-H., Kim S.-Y., An C.H., Lee S.H., Jung E.S., Park H.C., Kim M.S., Chung Y.-J., Lee S.-H. (2018). Clonal structures of regionally synchronous gastric adenomas and carcinomas. Clin. Cancer Res..

[24] Liu Y., Zhang J., Li L., Yin G., Zhang J., Zheng S., Cheung H., Wu N., Lu N., Mao X. (2016). Genomic heterogeneity of multiple synchronous lung cancer. Nat. Commun..

[25] Hanahan D., Weinberg R.A. (2011). Hallmarks of cancer: The next generation. Cell.

[26] Chou T.-Y., Dacic S., Wistuba I., Beasley M.B., Berezowska S., Chang Y.-C., Chung J.-H., Connolly C., Han Y., Hirsch F.R. (2025). Differentiating separate primary lung adenocarcinomas from intrapulmonary metastases with emphasis on pathological and molecular considerations: Recommendations from the International Association for the Study of Lung Cancer Pathology Committee. J. Thorac. Oncol..

[27] Yoo S.B., Chung J.-H., Lee H.J., Lee C.-T., Jheon S., Sung S.W. (2010). Epidermal growth factor receptor mutation and p53 overexpression during the multistage progression of small adenocarcinoma of the lung. J. Thorac. Oncol..

[28] Sartori G., Cavazza A., Bertolini F., Longo L., Marchioni A., Costantini M., Barbieri F., Migaldi M., Rossi G. (2008). A subset of lung adenocarcinomas and atypical adenomatous hyperplasia-associated foci are genotypically related: An EGFR, HER2, and K-ras mutational analysis. Am. J. Clin. Pathol..

[29] Wang C., Shen H. (2019). Commentary: Premalignant genomic data tracing the evolution of lung adenocarcinoma. EBioMedicine.

[30] Lee J., Han Y.B., Kwon H.J., Lee S.K., Kim H., Chung J.-H. (2022). Landscape of EGFR mutations in lung adenocarcinoma: A single institute experience with comparison of PANAMutyper testing and targeted next-generation sequencing. J. Pathol. Transl. Med..

